# The impact of generics and generic reference pricing on candesartan and rosuvastatin utilisation, price and expenditure in South Africa

**DOI:** 10.1007/s11096-018-0758-x

**Published:** 2018-11-26

**Authors:** Henk de Jager, Fatima Suleman

**Affiliations:** 1 Discipline of Pharmaceutical Sciences, School of Health Sciences, University of KwaZulu-Natal (Westville Campus), Private Bag X54001, Durban, KwaZulu-Natal 4000 South Africa; 2 Faculty of Science, Utrecht University, Utrecht, The Netherlands

**Keywords:** Candasartan, Generic reference pricing, Generics, Medicine expenditure, Rosuvastatin, South Africa

## Abstract

*Background* In the South African private sector context, generically similar products are grouped together and the reimbursement rate is set at the average price of the generically equivalent products. Very little evidence exists in low and middle-income countries with regards to the impact of this policy over time. *Objectives* To determine the impact of the introduction of generics and generic reference pricing on candesartan and rosuvastatin in the South African private health care sector in terms of medicine utilisation, medicine price and medicine expenditure. *Setting* South African private health sector. *Method* Medicine claims for candesartan and rosuvastatin was obtained from a Pharmacy Benefit Manager in South Africa. The claims covered a 48-month period from January 2012 to December 2015 and provided a pre- and post-reference price period for analysis. Medicine utilisation was measured as the number of Defined Daily Doses dispensed per 100,000 beneficiaries. Medicine price and expenditure was calculated as the average per Defined Daily Dose. *Main outcome measure* Medicine utilisation, price and expenditure. *Results* Candesartan experienced an average 7.0% year-on-year decline in utilisation and rosuvastatin a 5.0% increase. Medicine expenditure reduced by an additional 34.6% and 20.9% for candesartan and rosuvastatin respectively. The total savings was 54.8% for candesartan and 31.9% for rosuvastatin. *Conclusion* The introduction of generics and generic reference pricing did not have an impact on medicine utilisation, but reduced the price and expenditure of both candesartan and rosuvastatin.

## Impacts on practice

- Generic substitution and generic reference pricing could reduce expenditure and out of pocket payments by patients.
- Generic substitution and generic reference pricing could reduce medicine expenditure without affecting health outcomes.
- Utilisation of generic medicines, after patent expiry, improves over time as more competitors enter the market.

## Introduction

Pharmaceutical expenditure is still increasing internationally, although growth has slowed down since the mid-2000s but is on the rise again [1, 2]. In South Africa, pharmaceutical expenditure in the private sector reached ZAR 22.3 billion in 2015, a 36.8% increase from the ZAR 16.3 billion spent in 2012 [3]. The introduction of new medicines and the increasing demand for existing medicines are the main drivers of pharmaceutical spending [2]. The quantity of medicines consumed has increased over time in many therapeutic classes. Most notably, between 2000 and 2013, the use of antihypertensive medication in Organization for Economic Co-operation and Development (OECD) countries nearly doubled, while the use of cholesterol lowering drugs tripled [1]. In South Africa, a similar trend of increasing consumption can be seen, with hypertension prevalence increasing from 114.6 per 1000 beneficiaries in 2011 [3] to 152.8 per 1000 beneficiaries in 2015 [4]. Despite the nominal growth in pharmaceutical expenditure and the increased consumption, pharmaceutical expenditure as a percentage of total health expenditure has reached a plateau [1, 2]. In South Africa, pharmaceutical expenditure contributed 16.1% to overall health expenditure in the private sector in 2015, only marginally more than the 15.8% spent in 2012 [3].

Two factors that contributed to the stagnation in growth of pharmaceutical expenditure is the introduction of generic medicines and the introduction and strengthening of cost-containment policies [1, 5]. Generic medicines offer the opportunity to make substantial savings without affecting the quality of care, as has been evidenced on a study on cardiovascular medicines [6]. The introduction of generic medicines may lower utilisation of more expensive brand name products, which results in savings in pharmaceutical expenditure [2]. In South Africa, generic medicines are on average 22% cheaper than their brand name equivalents, and 56% cheaper than products without any generic competition [7]. Generic medicines were responsible for 56.2% of all drugs dispensed in South Africa in 2015, to beneficiaries of the medical schemes contracted with the Mediscor PBM [7]. One of the cost-containment policies used by medical schemes in South Africa, to promote the use of generic medicines is reference pricing. In essence, reference pricing groups therapeutically similar medicines together and sets a maximum reimbursement rate for the group of medicines [8]. Any medication with a price below or at the reference price will be covered in full, while medicine with a higher price will only be partially reimbursed with the beneficiary paying the balance between the price of the chosen medicine and the reference price. Reference pricing can change the demand for expensive brand name medicines, when people elect to use cheaper generic alternatives to avoid out-of-pocket co-payments. Reference pricing does not have adverse effects on health outcomes [8, 9]. It also does not increase the use of other health services, with the possible exception of an increase in doctors’ consultations when reference pricing is introduced, and patients want to switch to cheaper reference medicines [10].

### Background to the South African pharmaceutical environment

South Africa is a developing country with limited health care resources [11]. The country has a two-tiered health care system with 8.8 million people (16% of the population) insured by private insurers [3, 12]. A National Drug Policy (NDP) was established in 1996 which led to the establishment of a pricing committee and the introduction of a Single Exit Price (SEP) on all pharmaceuticals in the private health sector [13, 14]. Mandatory offer of generic substitution was introduced in 2003 which empowered pharmacists to offer to substitute brand name products with a generic equivalent [15, 16]. The Pharmaceutical Society of South Africa introduced a reference pricing model, called MMAP, or the Maximum Medical Aid Price, in 1985. Medical schemes could elect to pay only a specified maximum price for an off-patent product that had generic equivalents [17]. Versions of this model has been applied since that time by medical schemes in South Africa.

## Aim of the study

The aim of this study is to determine the impact of the introduction of generics and generic reference pricing on candesartan and rosuvastatin, which recently lost their patent protection, in an environment where generic reference pricing is already applied on other unprotected pharmaceutical products. The impact was measured on medicine utilisation, the average medicine price as well as the impact on medicine expenditure. This study will help provide some insight into the question regarding whether reference pricing has longer term benefits after the initial introduction of the reference pricing policy, which usually results in a reduction in medicine expenditure.

## Ethics approval

Ethical approval for this study was granted by the University of KwaZulu-Natal (UKZN) Biomedical Research Ethics Committee (BREC), reference number BE348/15.

## Method

This study is a retrospective longitudinal analysis of a medicine claims database. Medicine claims data was supplied by an independent Pharmacy Benefit Manager (PBM) in South Africa. For the study period, the PBM processed medicine claims for 1.45 million private health care beneficiaries in South Africa (about 18% of insured beneficiaries in South Africa). The database includes demographic information about the beneficiaries, including: date of birth, age, and gender. All beneficiary identification numbers were decoded and de-identified to ensure confidentiality. To ensure the reliability and validity of the results, a list of quality criteria for interrupted time series designs was adopted when the research methods and tools were designed [18].

The study period covered a 48-month period from January 2012 to December 2015. Both rosuvastatin and candesartan received generic competition in this period and generic reference pricing was subsequently introduced on the active ingredients. Generic reference pricing was introduced in April 2013 for rosuvastatin, and in February 2014 for candesartan. For both rosuvastatin and candesartan, the study period provided a pre-intervention base where generic reference pricing was not applied, to determine the impact of generic reference pricing after generic alternatives became available.

Only beneficiaries from registered medical schemes who were contracted with the PBM for the entire study period were included in the study population. This was done to control inconsistencies in the before and after comparisons of the introduction of generic reference pricing, because of changes in volume and the make-up of the study population. Of the medical schemes contracted with the PBM for the entire study period, only those who applied generic reference pricing were included in the study population. Medical schemes that had major benefit design changes in the study period, e.g. changes in medicine formulary or other co-payment changes, were excluded from the study. These changes in benefit design could have an impact on the utilisation and expenditure of rosuvastatin and candesartan, not because of the introduction of generics and generic reference pricing. Demographic information of the patients in the data sets before and after the introduction of reference pricing for the two therapeutic groups were analysed and compared.

Changes in medicine utilisation was measured by converting the claimed quantity to Defined Daily Dose (DDD) per 100,000 beneficiaries. DDD represents the assumed mean maintenance dose per day for a medicine when used for its main indication [19]. The standardisation of medicine volume to DDD enables utilisation comparisons across the different strengths of the same active ingredient. To control changes in the study population, the medicine volume was calculated as DDD dispensed per 100,000 beneficiaries, based on the membership data of the medical schemes included in the study population.

All medicine prices were obtained from the South African Medicine Price Registry, Database of Medicine Prices [20]. Prices are expressed in South African Rand (ZAR) and were applied for the year in which generic reference pricing was first introduced on the active ingredient. The Single Exit Price Adjustment (SEPA) published in the South African Medicine Price Registry was used to adjust the prices according to the year in which the product was dispensed [7], as products may apply for temporary or permanent reductions in a year, and these are not always captured in the database published at certain points in time on the website. Prices for candesartan are expressed in ZAR 2014 (Q2) and rosuvastatin in ZAR 2013 (Q1). Prices excludes dispensing fee and sales tax (value added tax (VAT) in South Africa). Price was calculated by multiplying the volume sold with the adjusted SEP and dividing by the number of DDD dispensed. Price therefore always refers to the price per DDD of the active ingredient. Medicine expenditure was calculated by multiplying the medicine price with the volume utilised and subtracting the patient out-of-pocket co-payments because of the application of generic reference pricing.

## Results

The characteristics of the 1444 beneficiaries using candesartan, and the 10,452 beneficiaries using rosuvastatin were stable (contracted during the entire study period) during the 48-month study period. For candesartan, 765 beneficiaries had claims in both the pre- and post-reference price periods, and for rosuvastatin 4738 beneficiaries claimed in both periods. Women represented 46% of the beneficiaries for the candesartan group and 43% of the rosuvastatin group. The mean age was 65.0 (standard deviation 12.3 years) and 61.2 years (standard deviation 11.5 years) for the candesartan and rosuvastatin groups respectively by the end of the study period.

### Medicine utilisation

**Fig. 1:**
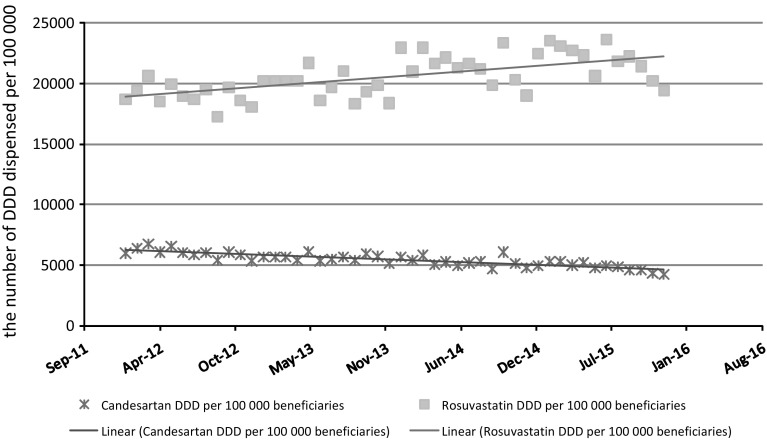
Candesartan and rosuvastatin utilisation for the period January 2012 to December 2015

Candesartan experienced a 19.6% reduction in DDD dispensed per 100,000 beneficiaries over the study period, or an average 7.0% year-on-year change over the 4 years. Rosuvastatin experienced a 15.6% increase in DDD dispensed per 100,000 beneficiaries over the study period, or an average 5.0% year-on-year over the 4 years. As illustrated in Fig. 1, the change in the number of DDD dispensed per 100,000 beneficiaries was a gradual change over time for both candesartan and rosuvastatin and was not caused by a big shift because of the introduction of generics and generic reference pricing.

**Fig. 2:**
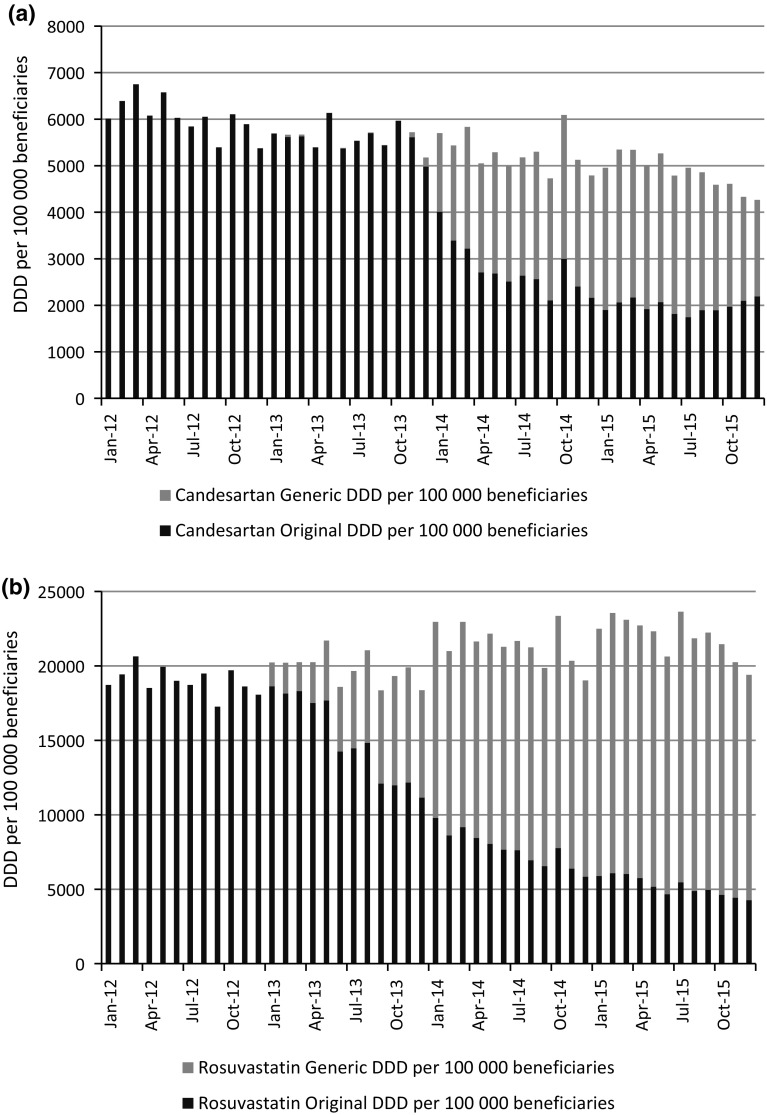
**a** Candesartan and **b** Rosuvastatin utilisation of original brand name product and generic alternatives, measured in DDD per 100,000 beneficiaries

Although the overall number of DDD dispensed per 100,000 beneficiaries was not affected by the introduction of generics and generic reference pricing, there was a notable change in the mix of original brand name products versus generic alternatives dispensed after the introduction of generics and generic reference pricing. The initial uptake of generic equivalents in the year of the introduction of generic reference pricing was also notable and increased even further in subsequent years. For candesartan, the generic utilisation reached 47.4% in the year of the introduction of generic reference pricing, and grew further to 59.3% in the following year. Generic utilisation of rosuvastatin started at 24.0% in the year of the introduction of generic reference pricing, and increased to 63.9% in the subsequent year and 76.4% in the year thereafter. Figure 2 illustrates the change in the mix of original brand name products and generic equivalents over the study period.

### Medicine price in terms of cost to schemes

Average price reductions range from 13.9 to 31.0% for rosuvastatin and candesartan respectively. The average price per DDD for candesartan reduced from ZAR 4.28 to ZAR 2.96, while the average price per DDD for rosuvastatin decreased from ZAR 8.06 to ZAR 6.94 in the study period. The magnitude of the difference in price between the original brand name product compared to the average price of the generic equivalents was much greater for candesartan compared to rosuvastatin. For candesartan, the average generic equivalent product is 54.5% cheaper than the original brand name product, while the difference in price for rosuvastatin was only 24.9% (Table 1).

**Table 1:** Candesartan and rosuvastatin price (in ZAR) and expenditure (in ZAR) per DDD

	Candesartan			Rosuvastatin		
	Pre RP^1^	Post RP^2^	Saving	Pre RP^3^	Post RP^4^	Saving
Average price per DDD	4.28	2.95	31.0%	8.06	6.94	13.9%
Original brand price per DDD	4.32	4.20	2.8%	8.08	8.20	− 1.6%
Average generic price per DDD	N/A^5^	1.91	N/A^5^	N/A^5^	6.16	N/A^5^
Average expenditure per DDD	4.28	1.93	54.8%	8.06	5.49	31.9%
Price saving per DDD		1.33	31.0%		1.12	13.9%
Reference price saving per DDD		1.02	34.6%		1.45	20.9%
Total saving per DDD		2.35	54.8%		2.57	31.9%

^1^The pre-reference price period for candesartan was from January 2012 to January 2014

^2^The post-reference price period for candesartan was from February 2014 to December 2015

^3^The pre-reference price period for rosuvastatin was from January 2012 to March 2013

^4^The post-reference price period for rosuvastatin was from April 2013 to December 2015

^5^There were no generic equivalent products available in the pre-reference price period. As a result, the average price per DDD and saving could not be calculated

### Medicine expenditure by the schemes

**Fig. 3:**
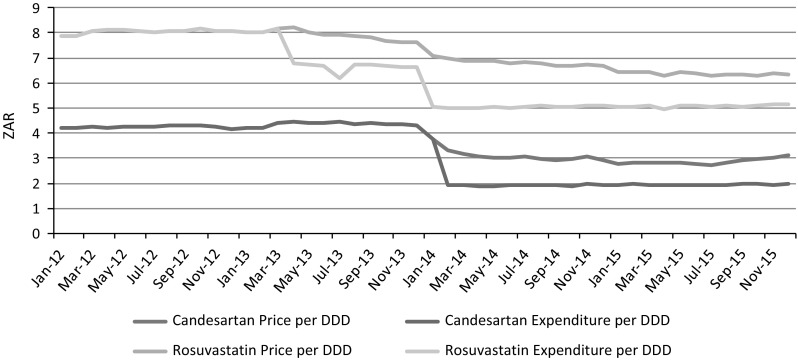
Candesartan and rosuvastatin price and expenditure per DDD by the schemes for the period January 2012 to December 2015. ^1^Generic reference pricing for candesartan was introduced in February 2014. ^2^Generic reference pricing for rosuvastatin was introduced in April 2013. ^3^In the period March 2013 to December 2013 there was only one generic equivalent product available for rosuvastatin, distributed by the same manufacturer as the original brand name product. ^4^The price per DDD reflects the arithmetic mean of the cost per DD to schemes, based on actual products, assumed (estimated) prices and actual volumes supplied, and the expenditure reflects the expenditure by the schemes after deduction of co-payments

The introduction of generic reference pricing produced an additional saving on medicine expenditure of 34.6% for candesartan and 20.9% for rosuvastatin. This saving is in addition to the 31.0% and 13.9% saving that resulted from the reduction in price per DDD because of the introduction of generic equivalents. Candesartan expenditure decreased from ZAR 4.28 to ZAR 1.93 per DDD after the intervention. Rosuvastatin expenditure decreased from ZAR 8.06 to ZAR 5.49 per DDD. The application of generic reference pricing resulted in a reference price co-payment of ZAR 1.02 per DDD for candesartan and ZAR 1.45 per DDD for rosuvastatin. Figure 3 illustrates the additional impact of generic reference pricing on medicine expenditure after the introduction of the reference price in February 2014 for candesartan, and April 2013 for rosuvastatin.

The total saving in medicine expenditure per DDD is 54.8% for candesartan and 31.9% for rosuvastatin. This equates to a saving of ZAR 1.4 million for candesartan and ZAR 8.8 million for rosuvastatin during the post-reference price period in the study, based on the membership data of the medical schemes included in the study population.

## Discussion

The introduction of generics and generic reference pricing had an impact on the mix of original brand name products and generic equivalents that were claimed in the post-reference price period. The higher generic uptake for rosuvastatin can be ascribed to a longer post-reference price period, greater number of generic competitors during this period, and the introduction of a clone product by the manufacturer of the original brand name product. The high initial generic uptake of both candesartan and rosuvastatin may be due to the practice of both generic substitution (since 2003) and generic reference pricing on other classes of pharmaceutical products.

The greater number of generics and generic uptake for rosuvastatin did not result in greater savings in price per DDD in the post-reference price period. The price per DDD for rosuvastatin decreased by 13.9%, while candesartan experienced a 31.0% reduction. The greater savings in price per DDD for candesartan may be a result of a greater price difference between the original brand name product and the average price of generic equivalents. For candesartan, the average price of the generic equivalent products was 54.5% cheaper than the brand name product, while for rosuvastatin the difference was only 24.9%. The price difference for rosuvastatin is however somewhat diluted, because for the first 6 months after the introduction of reference pricing there was only one generic equivalent product available, distributed by the same manufacturer as the original brand name product at a 19.7% discount. In the following 24 months, an additional three competitor generic equivalent products were launched, and the premium on the brand name product increased to 27.0% compared to the average generic price. The difference between the price of the original brand name product and the generic equivalents is not as big as seen in other countries in the world [1, 2, 10]. This could possibly be because of a smaller number of generic competitors in the South African market as well as the price controls enforced through the SEP legislation.

The reduction in the average price per DDD could be attributed to the introduction of generic alternatives. Although it is possible that reference pricing had an influence on the decision of beneficiaries to move to a generic alternative, this study cannot correlate the change directly to reference pricing because beneficiaries did not have the opportunity to choose a generic alternative before the introduction of reference pricing. Reasons for the decline in candesartan utilisation, and the increase in rosuvastatin utilisation might reflect substitution between members of each pharmacological class, perhaps influenced by the generic reference pricing policy. Prescribers might have switched to AII inhibitors that were not subject to reference pricing, or away from other statins where a clone option did not present itself. These would need to be explored further.

The introduction of reference pricing did, however, result in reductions in the expenditure per DDD because of the out-of-pocket co-payments experienced by members who elected to use a product above the reference price. It can be argued that this additional saving is only a saving to the insurer and not a saving to overall health care expenditure, because beneficiaries will be responsible for out-of-pocket co-payments. Further research is required as to how many beneficiaries opted for the additional out-of-pocket expenditure and why. Candesartan experienced an additional 34.6% saving on the expenditure per DDD because of the introduction of reference pricing, resulting in an overall saving of 54.8% per DDD. Rosuvastatin had an additional saving of 20.9% in expenditure per DDD, resulting in an overall saving of 31.9%.

Historically there are few published studies of the actual effect of reference pricing [21]. There remains, in low and/or middle-income countries (LMIC) a dearth of published research on the impact of pharmaceutical policies to increase the use of generics, including reference pricing, both at national and private insurer level [22, 23]. In a meta-analysis of published literature from 2000 to 2010, Kaplan et al. [23] referenced only one article on generic reference pricing in LMICs. In their conclusion they stressed that ‘Evaluations of generic medicines policies in LMICs are urgently needed’.

Unfortunately, the number of reference pricing categories analysed was limited and was only representative of one of several reference pricing systems in South Africa’s private sector. Nevertheless, this study adds to the evidence that is required to assess the impact of this policy on the impact of reference pricing on medicines utilisation and costs for South Africa.

## Conclusion

Generic reference pricing offers the ability to generate additional savings in pharmaceutical expenditure in the longer term, as more original brand name products lose their patent protection and generic alternatives are introduced in the market. This study only focussed on one part of the private health care market in South Africa, and on one reference pricing category, and the impact on the entire private sector still needs to be determined. Further studies are needed on more products as well as the impact on the entire therapeutic class to ensure that beneficiaries aren’t switching to other products not affected by reference pricing.
